# Nitrogen and sulfur dual doped porous carbon as metal-free catalyst for oxidative degradation of 4-nitrophenol by persulfate activation

**DOI:** 10.1038/s41598-023-28470-x

**Published:** 2023-01-21

**Authors:** Yasamin Bide, Niloofar Naseri Jahromi

**Affiliations:** Department of Chemical Technologies, Iranian Research Organization for Science and Technology (IROST), P.O. Box: 15815-3538, Tehran, Iran

**Keywords:** Environmental chemistry, Nanoscale materials

## Abstract

The replacement of metals in catalytic processes is highly demanded to improve sustainability and economic growth. Poor stability and metal leaching are the main drawbacks of metal-based catalytic reactions. This work represented the use of nitrogen and sulfur-co-doped mesoporous carbon material ((N, S)-MPC) as a metal-free catalyst for the degradation of 4-nitrophenol (4-NP) as a priority pollutant announced by the Environmental Protection Agency through the persulfate-based advanced oxidation process. A low amount of (N, S)-MPC catalyst (0.3 g/L) exhibited superior performance for the degradation of 4-NP within 3 h at room temperature and unadjusted pH. The COD removal was calculated to be 76% using (N, S)-MPC catalyst. Interestingly, the degradations kinetics of 4-NP followed the zero-order kinetics with the rate constant of 0.505 min^−1^. The radical quenching experiment was accomplished to investigate the activation pathway of degradation. A real sample from an oil and gas company was treated with the (N, S)-MPC catalyst, which showed excellent total decontamination of 61%. The recyclability and stability of the catalyst have been evaluated for three runs. Owing to the obvious benefits such as high efficiency, metal-free nature, and recyclability, the presented catalyst can improve pollutant removal from aqueous media and practical environmental remediation.

## Introduction

4-nitrophenol (PNP) is a toxic and persistent contaminant of industrial effluents, soil, and groundwater that is widely used in the synthesis of pesticides, herbicides, pharmaceuticals, and dyes^1,2^. It has been considered the priority pollutant in several countries resulting in potential risk to humans and the ecosystem even at low concentrations^3,4^. Thus, it is highly demanded to develop an efficient, cost-effective, and rapid technique to improve PNP degradation in aqueous environments. An advanced oxidation process (AOP) is considered a feasible approach to PNP removal^5–7^. Compared to the traditional AOP based on hydrogen peroxide, persulfate represents higher selectivity, lower reaction requirements, and milder conditions^8^.

Persulfate-based AOP establishes a significant development in wastewater treatment and environmental remediation^9^. Owing to the low oxidative potential, the persulfate alone shows a relatively slow reaction with pollutants^10^. Therefore, various activators such as thermal, ultrasound, radiation, alkaline, and metal activators have been employed to produce reactive radicals and achieve efficient pollutant removal^11^. The high energy consumption, high pH dependency, disposal of precipitates, metal agglomeration and leaching, resource scarcity, and toxicity of some metals are some drawbacks of common AOP activators. To overcome these problems, particularly secondary contamination, metal-free catalysts have recently emerged as one of the fastest-growing research fields^12,13^. In 2015, Duan et al.^14^ investigated the various dimension of carbon materials as metal-free catalysts for persulfate activation. According to their results, porous carbon material showed high activity due to the defects and pore structure. On the other hand, the introduction of heteroatoms is an operative approach to improving the performance of metal-free carbonaceous materials for persulfate activation^15,16^. Very recently, Liu et al. applied sulfur-doped ordered mesoporous carbons for persulfate-based PNP removal which showed improved activity due to the introduction of more strain and defects^17^. On the other hand, nitrogen doping can affect the charge distribution of neighboring carbon atoms, inducing Lewis acid character to graphitic carbons, structural defects, bond disorders, and lattice distortion in a conjugated carbon network^18^. The nitrogen and sulfur co-doping exhibits a synergistic effect and enhanced electronic properties resulting in improved catalytic activity in various processes^19,20^. Therefore, in this work, nitrogen and sulfur doped mesoporous carbon material has been utilized as metal-free catalyst for persulfate-based AOP. Because of the synergistic effect of dual doping and the 3D mesoporous structure, (N, S)-MPC was expected to represent high efficiency.

## Experimental

### Materials

Sodium hypochlorite solution (5% active chlorine) (NaOCl) was purchased from Sehat Industrial and Commercial Co. Acetic acid (glacial) 100%, 2-aminothiazole (2-AT), sodium peroxydisulfate, 4-nitrophenol (4-NP), sodium hydroxide, and potassium hydroxide were prepared from Merck Co. and used as received. The rest of the materials and solvents were obtained from Merck Co.

### Instruments and characterization

To investigate the functional groups of the samples, FTIR spectroscopy was accomplished using a Philips PU 9600 Serie. The reduction of 4-NP and disappearance of the yellow color were monitored by UV–Vis spectroscopy (SP8-400 UV/Vis Spectrophotometer). Field emission scanning electron microscopy (FESEM) was performed by a field emission microscope (Zeiss SIGMA VP, Oxford Instrument, 20 kV) equipped with energy dispersive X-ray spectroscopy (EDX) and a mapping detector (Oxford Instruments). To designate the Brunauer–Emmett–Teller (BET) surface area and pore size distribution, N_2_ adsorption–desorption isotherms were obtained using a BElSORP Mini apparatus (Microtrac Bel Corp). The samples were degassed in a vacuum at 200 °C for 8 h before N_2_ adsorption at − 195 °C. Raman pattern of (N, S)-MPC was provided using a Raman microscope (XploRA Plus, HORIBA) with a He/Ne Laser excitation at 532 nm. The chemical composition of the N, S-MPC was determined by using a CHNS elemental analyzer (LECO CHNS-932). The 4-NP concentration was analyzed by high-performance liquid chromatography (Agilent Technologies, Palo Alto, USA) with an ODZ-3 MZ column and UV detector. The mobile phase included acetonitrile (60%) and water (40%). The column temperature in HPLC analysis was 30 °C. The injection volume of the sample was 20 μl and the flow rate was 0.8 ml/min. The GC analysis was performed on the real sample before and after 24 h of AOP reaction using a PHILIPS gas chromatography device (PU4500 model) equipped with an FID detector. The samples were extracted three times with dichloromethane, dried with magnesium sulfate, and concentrate to a determined amount. Before the injection into the GC, the samples were filtered with a 0.45 μm filter. The 2 µL of the sample was injected into the GC. The initial column temperature was adjusted at 50 °C, followed by increasing to 240 °C at a rate of 5 °C/min, and kept for 5 min. The inlet temperature and detector temperature were set at 300 and 350 °C, respectively. The sample was injected into the GC column in the split-mode at a split ratio of 1:4.

### Synthesis of nitrogen and sulfur-co-doped mesoporous carbon material

To prepare N and S-co-doped mesoporous carbon material, the hydrothermal carbonization of poly(2-aminothiazole) (P(2-AT)) was conducted by a procedure described previously^21^, with some modifications. The polymerization of 2AT was simply performed using sodium hypochlorite as the oxidant and acetic acid as the dopant. The 50 mL aqueous solution of 0.26 M 2AT was mixed with 50 mL of 0.26 M acetic acid solution. The solution was rapidly mixed with 50 mL of 0.13 M sodium hypochlorite solution and then kept at 5 °C without shaking. Before mixing, two aqueous solutions should be reached at 5 °C. After 24 h, the brown powder was collected, centrifuged, and washed with 0.1 M KOH aqueous solution and DI water to remove unreacted monomers and oligomers and the excess reactants. Then, a specified concentration of P(2-AT) aqueous solution was prepared and moved to a Teflon-lined stainless-steel autoclave for hydrothermal carbonization at 180 °C for 12 h. The dark brown powder was washed with DI water and dried in an oven at 45 °C for 12 h.

### Persulfate-based advanced oxidation process of 4-nitrophenol using (N, S)-MPC

The persulfate-based AOP of 4-NP was examined using as-prepared (N, S)-MPC as the activating agent of sodium peroxydisulfate. In a typical procedure, 10 mL of 15 mg/L aqueous solution of 4-NP, 0.015 g sodium peroxydisulfate, and 0.003 g (N, S)-MPC were mixed in a round-bottom flask equipped with a magnetic stirrer. The mixture was centrifuged for 3 min to disperse the catalyst in the solution and then allowed to react for 3 h at 25 °C at unadjusted pH (pH = 6). After the completion of the reaction, the mixture was centrifuged to isolate the catalyst. The catalyst was washed with DI water and then dried for recyclability tests. The amount of 50 µl of 0.1 M NaOH solution was added to 4 mL of the remaining solution and then employed for HPLC or UV–Vis measurements. Various tests were conducted to investigate the effect of different parameters such as temperature, catalyst content, and sodium peroxydisulfate content.

## Results and discussion

### Synthesis and characterization of metal-free catalyst

The P(2-AT) has been synthesized by the chemical oxidation method using various oxidants and dopants as well as different procedures^22,23^. In this work, our attempt to synthesize P2AT with copper chloride and ferric chloride as the oxidant led to metal trapped in the polymer structure. Owing to the nitrogen and sulfur heteroatoms in the polymer structure, the complete removal of these metals seems impossible. As Ciftci et al. reported the existence of the Fe element in the polymer matrix is based on the elemental analysis after the polymerization of 2AT^24^. To ensure the metal-free nature of the as-prepared catalyst, among the various synthetic procedures, we chose the rapid mixing polymerization of 2AT using acetic acid as the dopant and sodium hypochlorite as the oxidant described previously with some modifications^25^. To enhance the polymerization yield, the reaction time was extended to 24 h, but to obtain the desired morphology, the polymerization was carried out at 5 °C.

**Figure 1:**
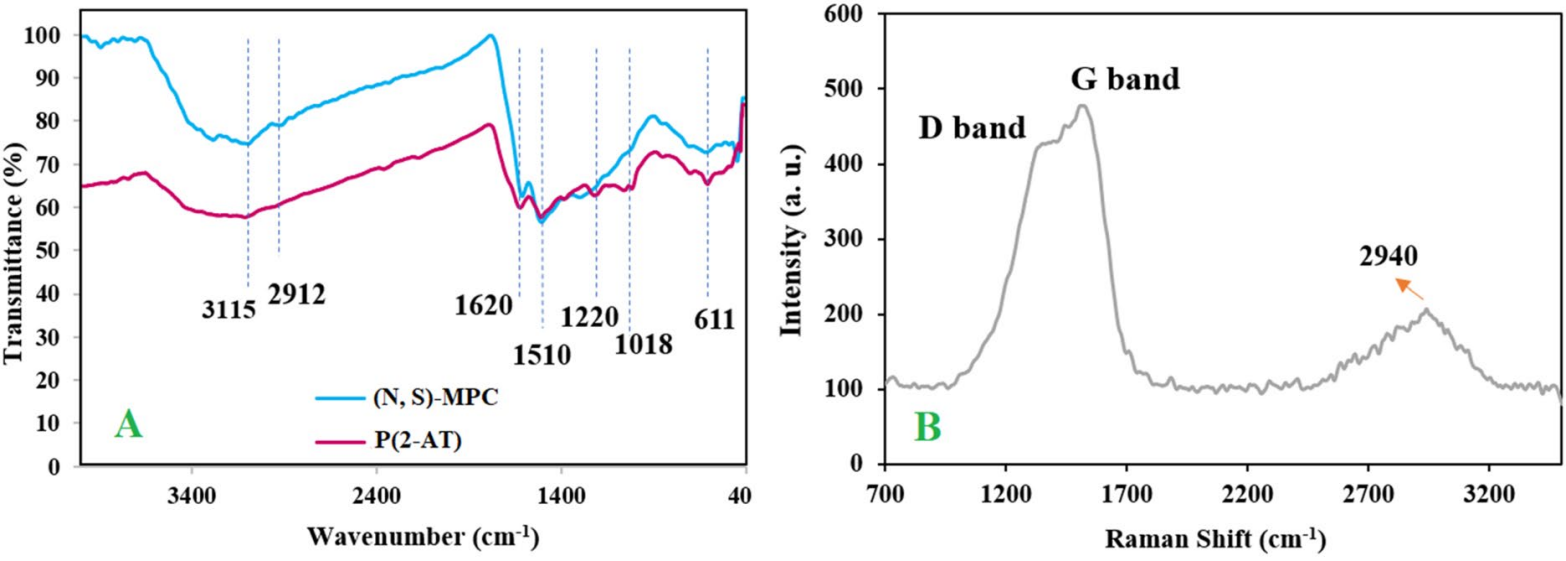
FT-IR spectra of P(2-AT) and (N, S)-MPC (**A**) and Raman spectrum of (N, S)-MPC (**B**).

To study the chemical structure and functional groups of as-synthesized P(2-AT) and (N, S)-MPC, FT-IR analysis has been carried out. FT-IR spectra of P(2-AT) were given in Fig. 1A. The peak at 1620 and 1510 cm^−1^ were related to the stretching vibrations of C=N and C=C in the polymer chain, respectively. The band at 3115 cm^−1^ was attributed to the stretching vibration of secondary –NH– groups demonstrating the formation of secondary –NH– linkages in P(2-AT) which is in good agreement with the literature^24^. The bands at 611 and 1220 cm^−1^ corresponded to C–S and C–N stretching vibrations, respectively. In the FT-IR spectrum of (N, S)-MPC (Fig. 1A), the peaks at 3115, 1620, and 1510 cm^−1^ showed an increase in their signals. Moreover, the C-N peak was shifted from 1220 to 1236 cm^−1^, probably due to the aromatization after hydrothermal treatment of P(2-AT).

The nitrogen and sulfur contents of N, S-MPC were determined by CHNS analysis as 9.4 and 23.66%, respectively. To investigate the structural and electronic properties of (N, S)-MPC, Raman spectroscopy analysis was carried out. As observed in Fig. 1B, the sample showed a G band at 1525 cm^−1^ and a D band at 1380 cm^−1^ related to the graphitic carbons^26^, as well as a peak at 2940 cm^−1^ ascribed to the 2D band. The G band is ascribed to the graphitic carbon with sp^2^ configuration and the D band indicates the defects in the carbon structure^27^. The heteroatom doping considerably enhances the defect density, which causes the strong D band and high intensity between the D and G bands (valley region). Ayiania et al. suggested that nitrogen functional groups significantly affect the vibrational modes in the valley region^28^. Therefore, the existence of the valley region also confirmed the heteroatom doping in the as-obtained porous carbon material.

**Figure 2:**
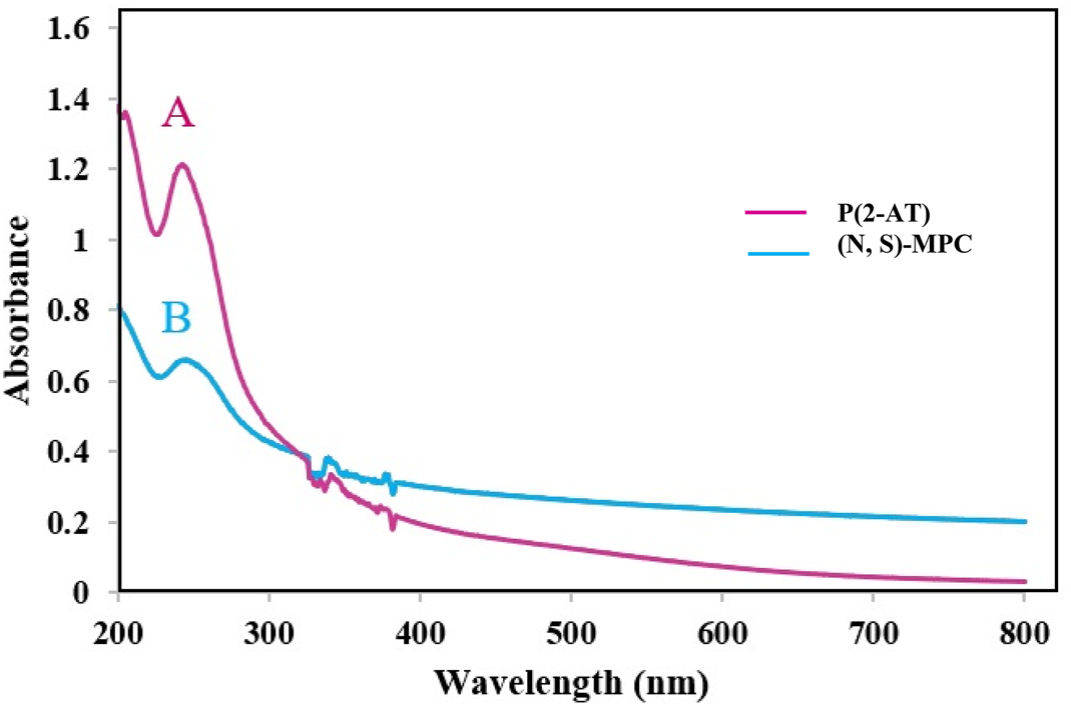
UV–Vis spectra of P(2-AT) (**A**) and (N, S)-MPC (**B**).

Figure 2 exhibited the UV–vis spectra of P2AT and (N, S)-MPC. Both of the spectra showed the peak at 248 nm ascribed to the π → π* transition of the conjugated π-system^29^. The lower absorbance after hydrothermal carbonization can be due to variations in the surface chemistry of the two types of nanoparticles.

**Figure 3:**
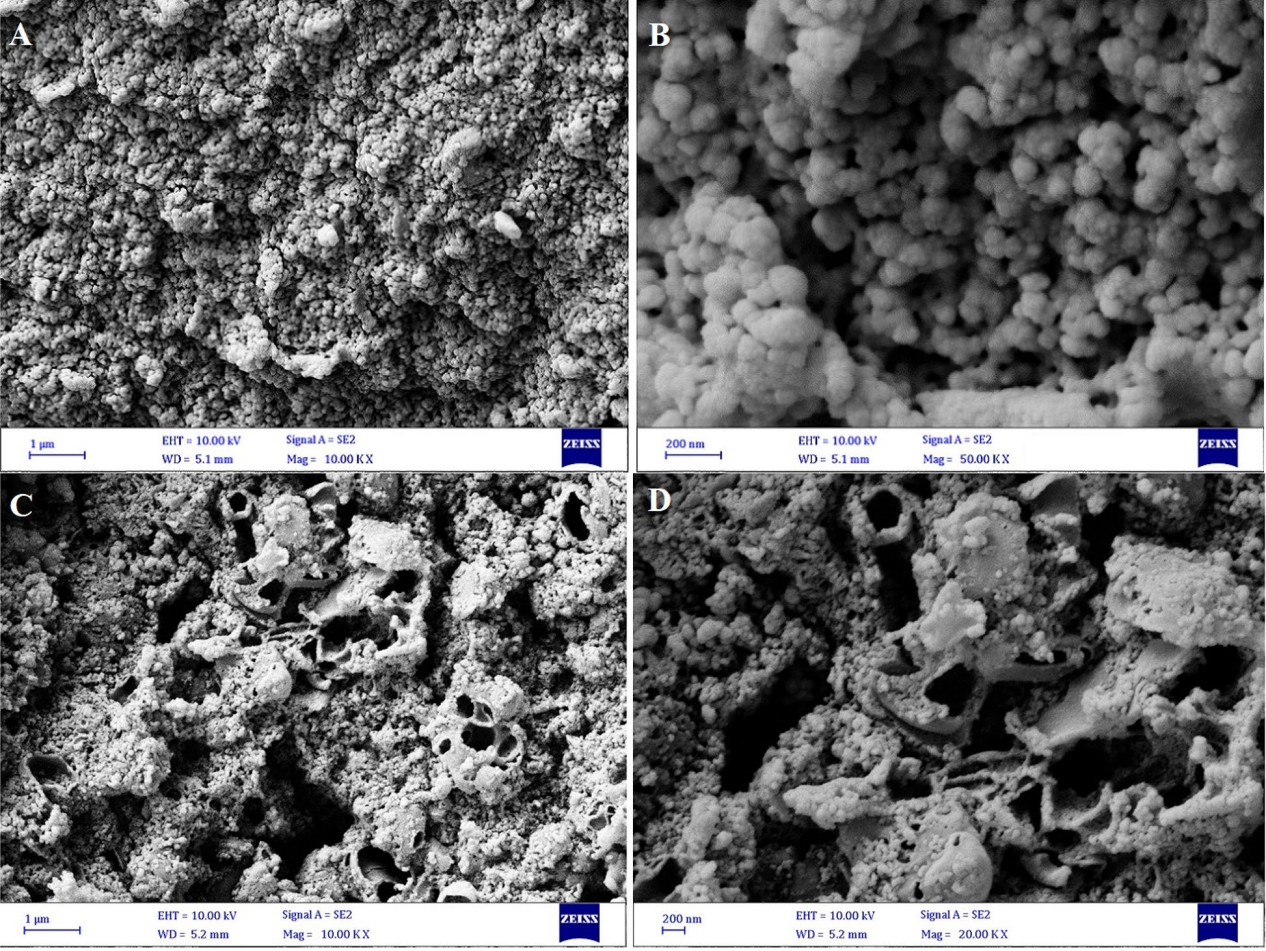
FT-IR spectra of P(2-AT) (**A**, **B**) and (N, S)-MPC (C, **D**).

**Figure 4:**
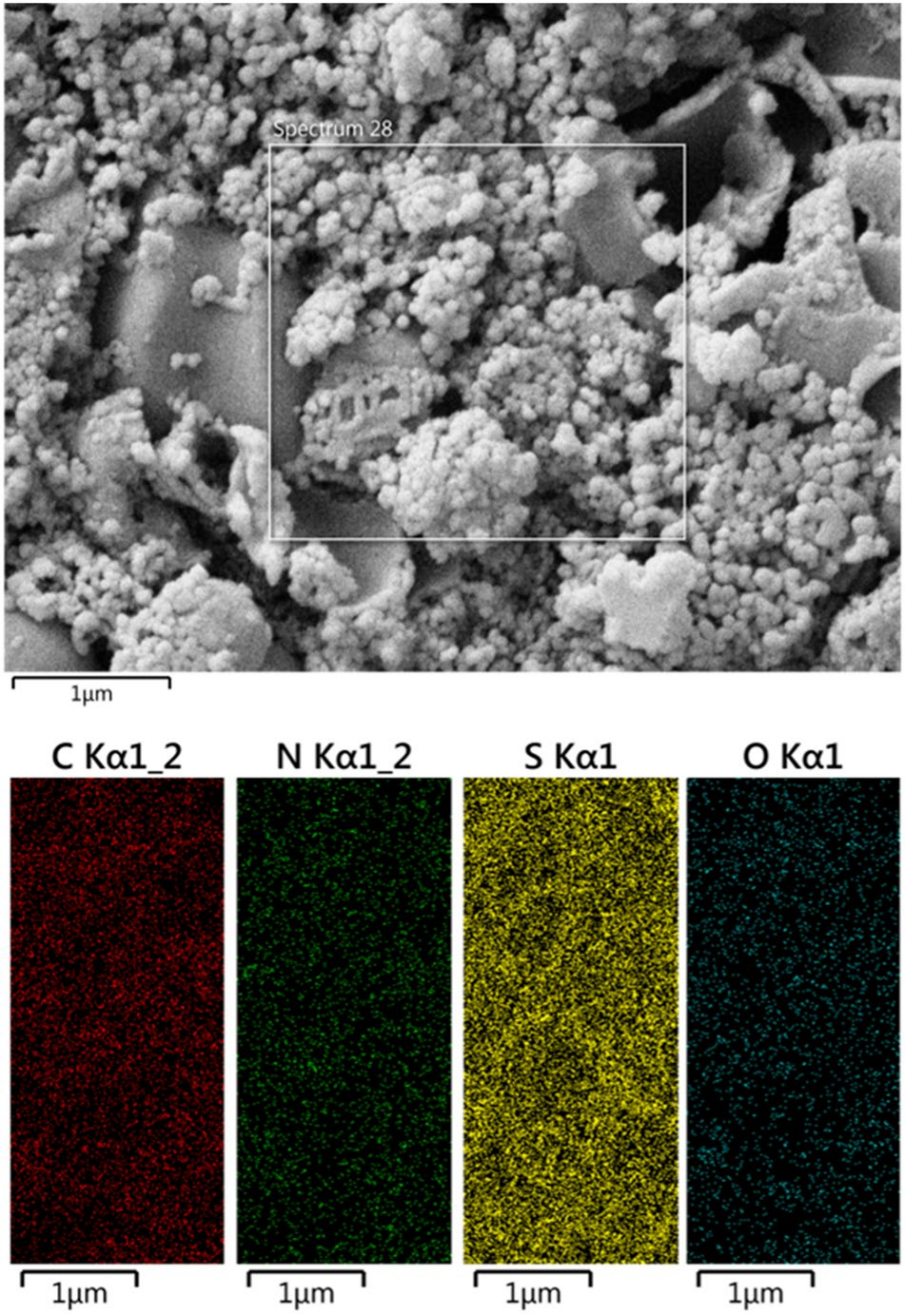
EDX elemental mapping of (N, S)-MPC.

The FESEM images explicitly designate that P(2-AT) possesses relatively uniform nanosphere morphology with an average particle size of < 100 nm (Fig. 3A and B). The hydrothermal treatment of as-synthesized P(2-AT) led to a porous material, as clearly seen in the SEM images of (N, S)-MPC. The EDX mapping analysis revealed the presence of carbon, nitrogen, sulfur, and oxygen in the (N, S)-MPC (Fig. 4). The matched spatial distributions of S, N, and C indicate the uniform distribution of sulfur and nitrogen within the carbon matrix.

**Figure 5:**
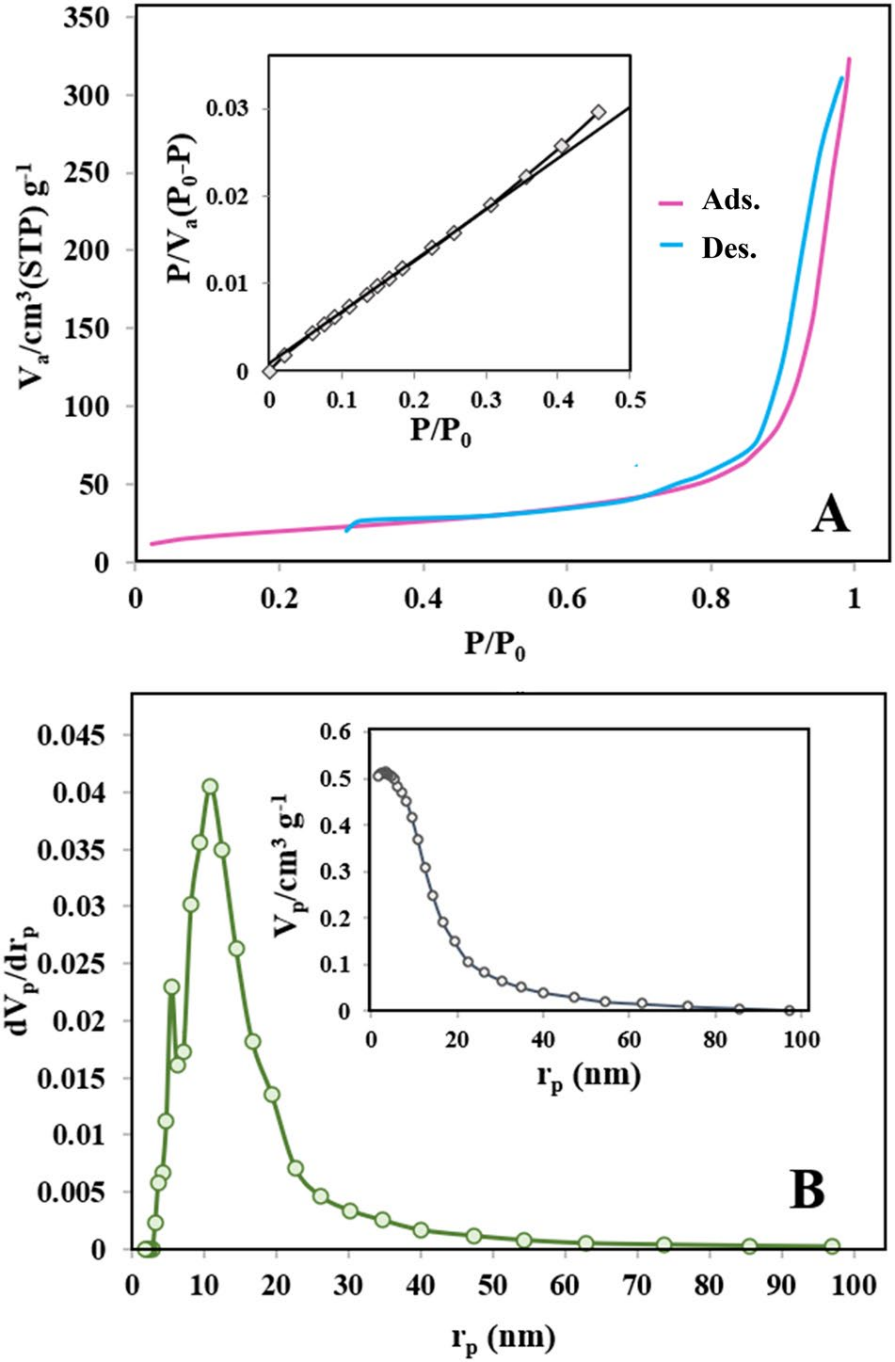
The nitrogen adsorption and desorption isotherm curves of (N, S)-MPC, inset: BET surface area plot (**A**), pore size distribution plot, inset: BJH adsorption cumulative pore volume (**B**).

The architecture of the porous structure of as-synthesized (N, S)-MPC was investigated by nitrogen adsorption and desorption measurements. The isotherms curve of (N, S)-MPC and BET plot (inset) are presented in Fig. 5A. The (N, S)-MPC indicated Type IV isotherm based on the IUPAC classification of isotherms which is related to the mesoporous materials^30^. Various mesoporous industrial adsorbents showed this isotherm, of which the initial monolayer-multilayer adsorption on the mesopore walls and pore condensation. The pore morphology can be identified by the type of hysteresis loop. The (N, S)-MPC showed an H1 hysteresis loop in which the vertical and parallel branches over a wide range of gas uptake are observed and abruptly increased at high pressures. The H1 hysteresis can be attributed to porous materials, including a narrow range of mesopores where the neck of the pores is only slightly narrowed^31^. This result is in good agreement with the SEM images (Fig. 3C and D), where the pores morphology can be detected. The pore size distribution and BJH cumulative pore volume (inset) have been given in Fig. 5B. The pore size distribution curve of (N, S)-MPC establishes that the pore size is distributed between 3.2 and 85.6 nm and mostly between 3.2 and 19.4 nm. The BET surface area equals 73 m^2^ g^-1^, the total pore volume equals to 0.50 cm^3^ g^-1^, and the average pore diameter equals 10.9 nm.

### Catalytic activity for persulfate-based AOP

**Figure 6:**
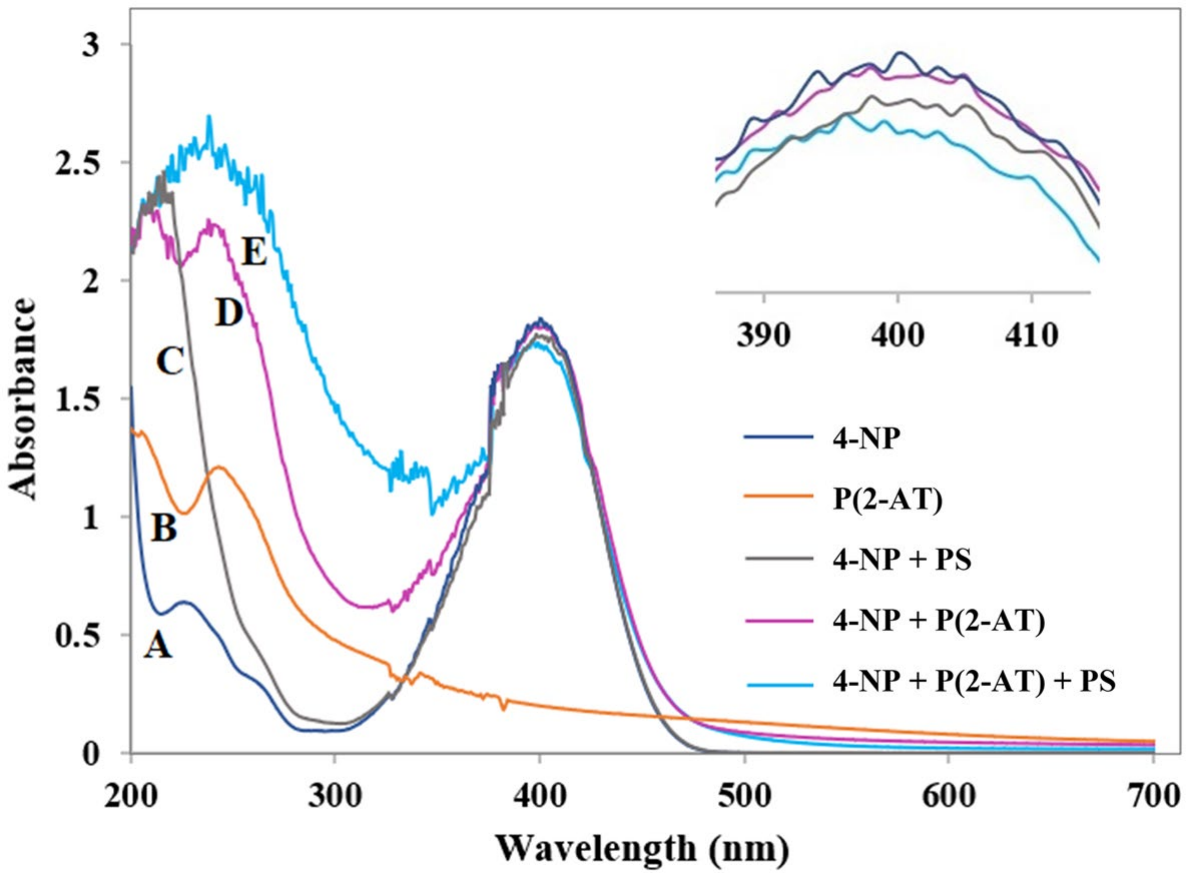
UV–Vis of an aqueous solution of 4-NP (**A**), P(2-AT) (**B**), 4-NP treated with persulfate after 3 h (**C**), 4-NP treated with P(2-AT) after 3 h (**D**), 4-NP after P(2-AT)-based AOP after 3 h (**E**), catalyst: 0.003 g, persulfate: 0.0015 g, 25 °C.

To evaluate the possible activity of as-synthesized P(2-AT) for 4-NP removal either with absorption or AOP mechanisms, various experiments have been accomplished and 4-NP removal has been monitored by UV–vis analysis. The UV–vis spectra of 4-NP and P(2-AT) have been given in Fig. 6A and B, respectively, as the control spectra. The AOP of 4-NP in the absence of catalyst was examined, indicating no conversion yield after 3 h (Fig. 6C). Moreover, the absorption of 4-NP by P(2-AT) was investigated, which showed 6% of 4-NP absorption after 3 h (Fig. 6D). The catalytic activity of P(2-AT) for persulfate-based AOP of 4-NP was tested that exhibited a low conversion yield (10%) with 0.015 g sodium peroxydisulfate (Fig. 6E). Based on the results, P(2-AT) did not show efficient absorption or AOP-based removal of 4-NP.

The catalytic activity of (N, S)-MPC as the metal-free catalyst for persulfate-based AOP of 4-NP was optimized using various amounts of catalyst and sodium peroxydisulfate. The results were summarized in Table 1. Increasing the catalyst amount from 0.001 to 0.003 considerably affected the efficiency of 4-NP removal. But, more increments from 0.003 to 0.006 led to a decrease in catalytic activity with a low slope. Since the best results have been obtained by 0.002 and 0.003 g catalyst, the sodium peroxydisulfate content has been optimized using these amounts. For 0.002 g catalyst, employing a 0.02 g sodium peroxydisulfate gave a 94% conversion yield (Table 1, entry 4). In the case of a 0.003 g catalyst, the reaction proceeded to 98% using 0.015 g sodium peroxydisulfate (Table 1, entry 7).

**Table 1:** Influence of the amount of catalyst and sodium peroxydisulfate on the degradation of 4-NP after 3 h at 25 °C.

Entry	Catalyst amount (g)	Sodium peroxydisulfate amount (g)	Conversion yield (%)
1	0.001	0.015	0
2	0.002	0.01	68
3	0.002	0.015	85
4	0.002	0.02	94
5	0.002	0.03	86
6	0.003	0.01	68
7	0.003	0.015	98
8	0.003	0.02	94
9	0.004	0.015	90
10	0.005	0.015	89
11	0.006	0.015	86

In addition, the effect of reaction temperature on the removal efficiency of the as-prepared catalyst was studied. The AOP reaction of 4-NP using 0.003 g catalyst and 0.015 g sodium peroxydisulfate was tested at 25, 40, and 55 °C. Based on the results, increasing the temperature from 25 to 40 °C led the reaction to complete after 60 min, and increasing the reaction temperature to 55 °C caused the reaction completion after 45 min.

Chemical oxygen demand (COD) specifies the degree of organic contamination, which is an important indicator of water treatment^32^. The efficiency of (N, S)-MPC catalyst for COD removal was also determined. The initial COD of 15 mg/L 4-NP was measured to be 37 mg/L. Due to the interference of persulfate for COD measurements and the overestimation, the corrected values were determined^33^. The COD of the reaction mixture without 4-NP (0.003 g catalyst, 0.015 g sodium peroxydisulfate, 10 mL water) as the blank sample was obtained to be 122 mg/L. On the other hand, the COD of the reaction mixture containing 10 mL 15 mg/L 4-NP, 0.003 g catalyst, and 0.015 g sodium peroxydisulfate after 3 h was determined as 131 mg/L. So, the corrected value of COD to eliminate the interference of persulfate was calculated as 12 mg/L. Comparing the initial COD and the corrected COD of the reaction mixture after 3 h, the COD removal equals 76%.

Several previously metal-free carbon-based materials for persulfate activation have been compared with this work in terms of synthetic procedure, catalyst amount, persulfate concentration, and removal efficiency. As given in Table 2, most of the reported heteroatom-doped carbon materials used as the metal-free carbon-based material for persulfate activation have been synthesized through heating at high temperatures under an inert atmosphere. Using a straightforward synthetic procedure to produce N, S-doped mesoporous carbon material for efficient pollutant removal is an outstanding advantage of this work.

**Table 2:** Comparing the performance of various metal-free carbon-based materials for persulfate activation.

Entry	Heteroatom-doped carbon material	Synthetic procedure	Catalyst amount	Persulfate concentration	Pollutant	Removal efficiency	Ref.
1	N-doped CNT	pristine CNT, nitrogen precursor, 750 °C for 1 h	100 mg/L	[PMS] = 0.2 mM	[Phenol] = 0.1 mM	95.6% t = 10 min	^18^
2	N-doped graphitic biochar	wetland plants (reed), ammonium nitrate, 900 °C for 90 min	20 mg	1.25 mL 160 mM	5 mL 1000 mg/L Orange G	100% t = 60 min	^34^
3	B, N-co-doped CNT	melamine, boric acid and CNT, 650 °C for 30 min	200 mg/L	[PMS] = 2.0 mM	[2,4-dichloro-phenoxyacetic acid] 0.1 mM	68% t = 240 min	^35^
4	Edge-enriched N, S doping	MWCNT and thiourea, 900 °C for 2 h	100 mg/L	[PDS] = 1.5 mM	BPA concentration = 20 mg/L	100% t = 30 min	^36^
5	N, S-co-doped carbons	Pyrolysis of sulfur-modified chitosan precursor, 750 °C for 2 h	200 mg/L	[Potassium persulfate (PS)] = 0.5 mM	[SMX] = 20 mg/L	98.62% T = 90 min	^37^
6	COF-derived hierarchically porous N-doped carbon	Terephthalaldehyde and melamine to obtain COFs by solvothermal method, carbonization at 700 °C for 2 h under N_2_	200 mg/L	[PS] = 20 mM	[2,4-dichlorophenol] = 60 mg/L	100% t = 40 min	^38^
7	biomass-derived N-doped porous carbon	Yeast extract and sodium bicarbonate solution heated at 80 °C overnight. Then, pyrolysis at 700 °C for 3 h	100 mg/L	[PS] = 6.5 mM PS	20 mg/L SMX	100% t = 20 min	^39^
8	nitrogen self-doped hierarchical porous carbon	carbonization-KOH activation process (800 °C for 3 h, 900 °C for 2 h) assisted by rapid-freezing technology using chitosan	200 mg/L	[PMS] = 0.2 g/L	[BPA] = 200 mg/L	100% t = 10 min	^40^
9	N,S-co-doped carbon	camphor sulfonic acid and melamine, 750 °C for 2 h	200 mg/L	[PS] = 0.4 mM	[SMX] = 20 mg L-1	96% t = 50 min	^41^
10	sulfur-doped CO_2_ converted carbon	molten salt carbon capture electrochemical transformation at 550 °C for 12 h under Ar	50 mg/L	PS/2,4-DCP = 5:1(mole ratio)	[2,4-dichlorophenol] = 20 mg/L	96.3% 150 min	^42^
11	S-doped activated carbons	chemical activation of polythiophene with KOH, annealing at 800 °C	50 mg/L	[PS] = 8 mM	[4-chlorophenol] = 40 mg/L	100% 60 min	^43^
12	(N, S)-MPC	Rapid mixing polymerization followed by hydrothermal carbonization at 180 °C for 12 h	300 mg/L	[PDS] = 1.5 g/L	[4-NP] = 15 mg/L	98% 180 min	This work

### Mechanistic study

To investigate whether the absorption mechanism is effective in the experiments, a test without persulfate was also accomplished, which indicated less than 10% 4-NP removal after 3 h. Therefore, two possible mechanisms have been considered, including the radical pathway and the non-radical pathway. The hydroxyl radicals are the main active species in the traditional AOP, while sulfate and hydroxyl radicals as well as singlet oxygen as nonradical species, can be dominant in persulfate-based AOP^44^. To determine the reactive oxidative species, three experiments were done using t-butanol, methanol, and benzoquinone as radical scavengers. Methanol efficiently quenches both ${\text{SO}}_{4}^{ \cdot - }$ and $^{ \cdot } {\text{OH}}$ , while t-butanol can efficiently scavenge $^{ \cdot } {\text{OH}}$ (3.8–7.6 × 10^8^). The persulfate-based AOP of 4-NP accomplished in the presence of 20 µL methanol showed a conversion yield of 6.6% after 1, 2, and 3 h reaction time. In the case of t-butanol, the conversion yield of 6.9% was obtained after 1 h, which remained constant. Very low conversions of 4-NP in the presence of two radical scavengers established the possibility of a radical mechanism. The comparable inhibition by the two scavengers indicated that $^{ \cdot } {\text{OH}}$ may be the main radical responsible for the 4-NP degradation in our system. Moreover, the benzoquinone was used as superoxide radicals (O_2_˙ˉ) scavenger, which is diminishes the 4-NP removal to 89% after 3 h, suggesting that O_2_˙ˉ is slightly involved in activation.

**Figure 7:**
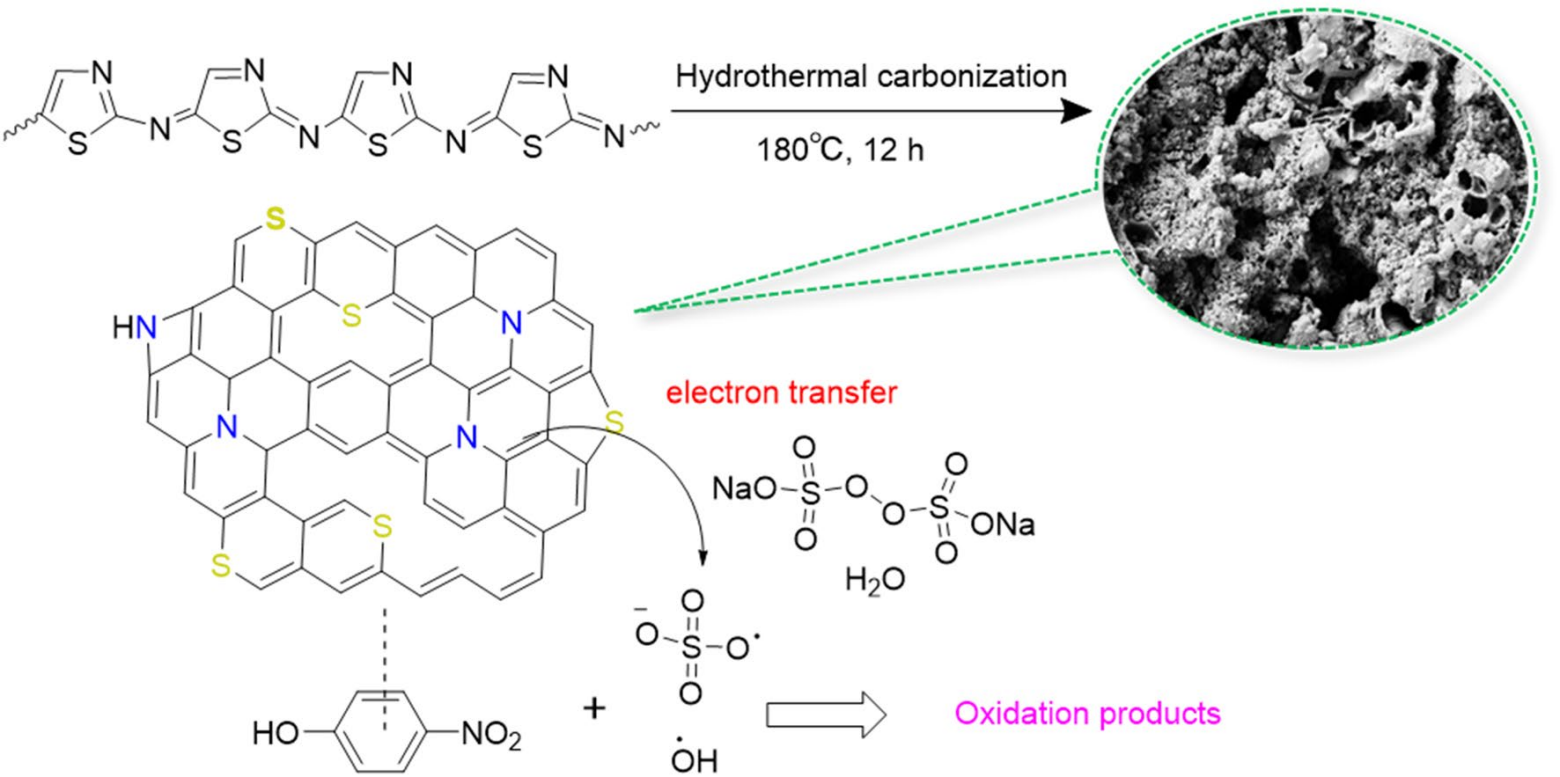
Schematic representation of 4-NP degradation using (N, S)-MPC as metal-free activator for persulfate-based AOP.

Several factors can be affected the efficient degradation of 4-NP using (N, S)-MPC as the persulfate activator (Fig. 7): (i) The nitrogen atoms with lone-pair electrons in the (N, S)-MPC can act as Lewis basic sites and induce the enhancement in the redox process; (ii) The higher electronegativity of nitrogen atoms causes the change of electronic structure of adjacent carbon atoms and enhancement of the electron transfer and consequently enabling sites for the surface reactions; (iii) Sulfur atoms with similar electronegativity to carbon but larger atomic size led to structural defects of the carbon network and consequently more active sites^45^; (iv) Sulfur atoms owing the lone pair can render electrons in the persulfate activation; (v) the carbon structure can act as electron bridge to simplify the electron-transfer; (vi) the carbon network can interact and activate the benzene ring of organic pollutants via π–π interaction which is beneficial for ring-opening process and synergistically enhance the oxidation by the reactive radicals.

### Kinetic study

The zero-order and pseudo-first-order degradation kinetics are usually employed in the heterogenous AOP catalysis system. The corresponding equations for zero-order and pseudo-first-order are given in Eqs. 1 and 2, respectively.

1 $$ {\text{C}}_{0} - {\text{ C}}_{{\text{t}}} = {\text{ k}}_{{{\text{app}}}} {\text{t}} $$

2 $$ - {\text{ln }}\left( {{\text{C}}_{{\text{t}}} /{\text{C}}_{0} } \right) \, = {\text{ k}}_{{{\text{app}}}} {\text{t}} $$

where C_0_([4-NP]_0_) and Ct ([4-NP]_t_) are the concentrations at the initial time, t = 0 and time “t”, k_app_ is the first-order rate constant (min^−1^).

**Figure 8:**
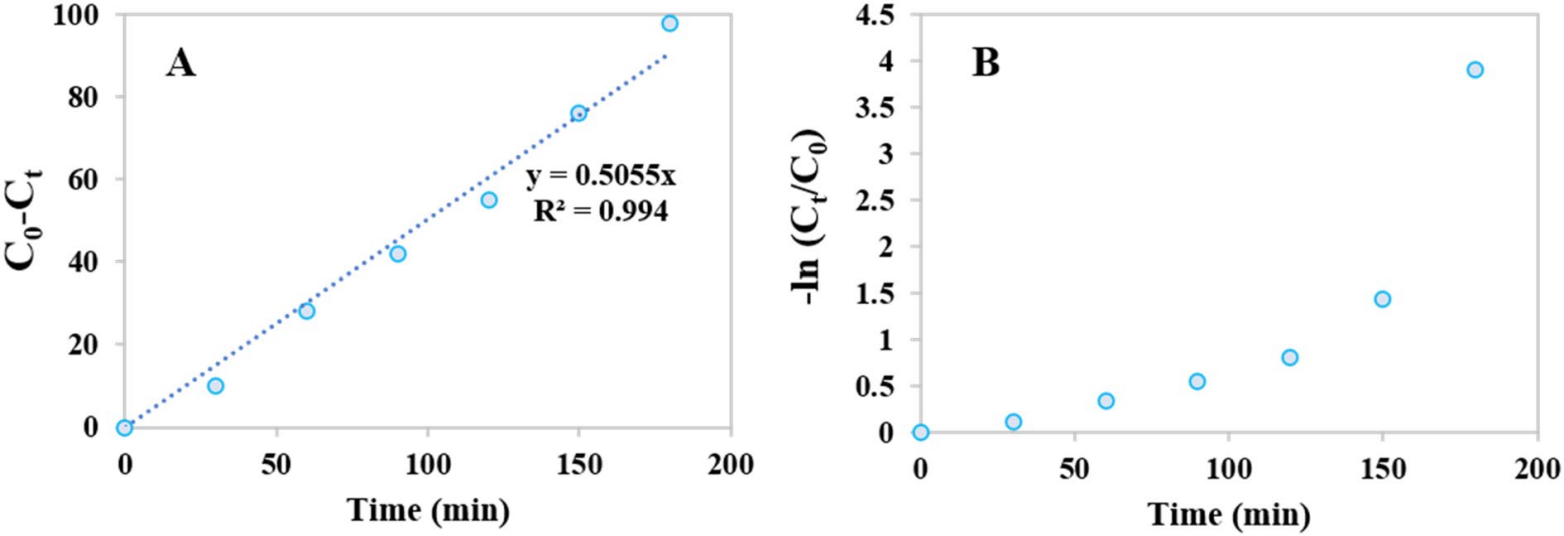
Zero-order (**A**) and pseudo-first-order (**B**) degradation kinetics of 4-NP on (N, S)-MPC.

The possible zero-order and pseudo-first-order degradation kinetics of 4-NP on (N, S)-MPC have been given in Fig. 8A and B, respectively. According to the fitting results, the degradations kinetics of 4-NP by (N, S)-MPC fitted pretty well with Eq. (1) (R^2^ = 0.994) (Fig. 8A) with a k_app_ of 0.505 min^−1^, which confirmed these degradation reactions might follow the Langmuir–Hinshelwood mechanism based on the reaction of adsorbed contaminant molecule with radicals formed on catalyst surface.

### Real sample application

**Figure 9:**
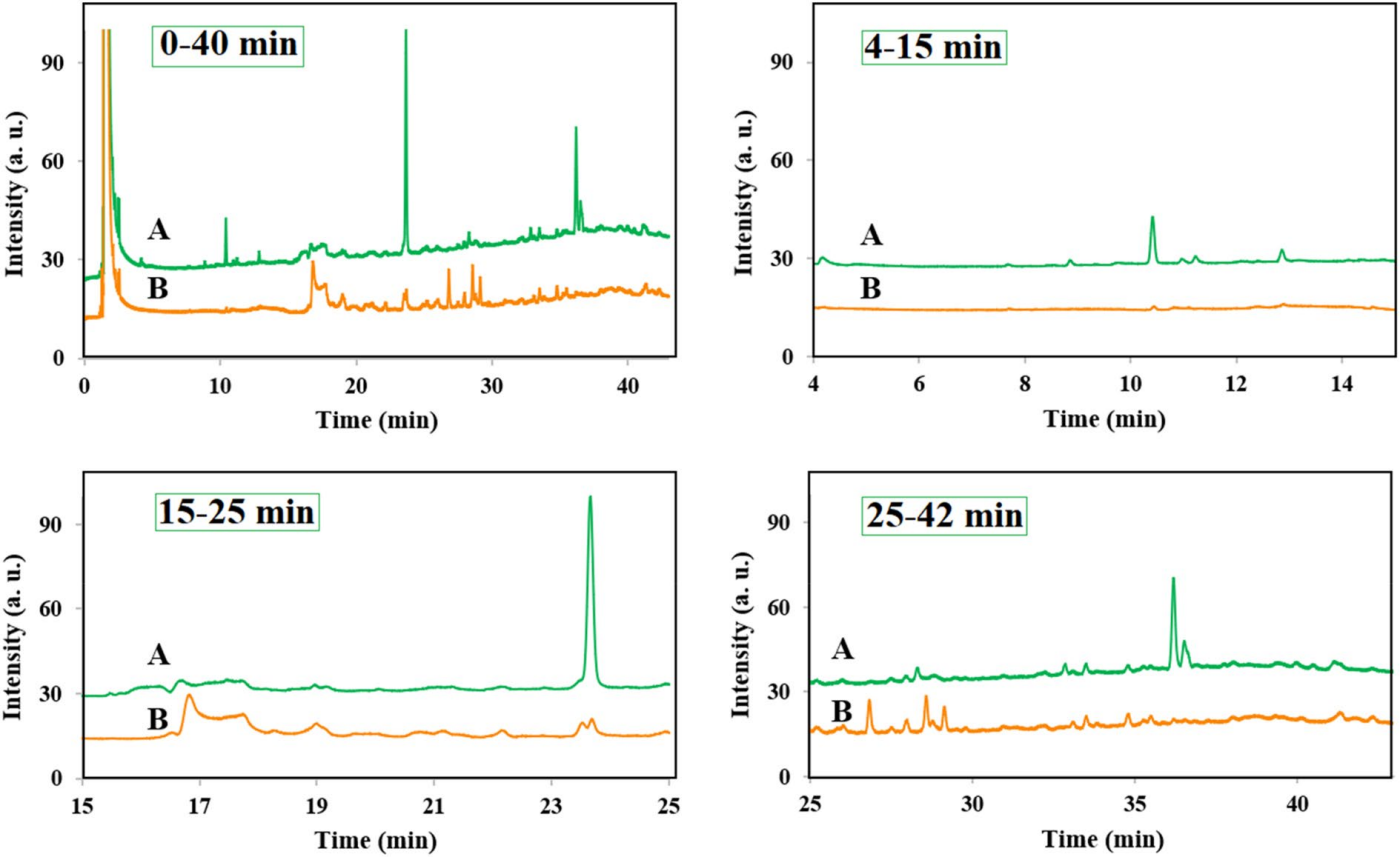
GC analysis spectra of real produced water sample before (**A**) and after (**B**) AOP treatment using (N, S)-MPC catalyst at four retention time regions including 0–40, 4–15, 15–25, and 25–42 min.

The catalytic activity of the as-synthesized metal-free catalyst has been examined for the AOP process and decontamination of real sample to indicate the applicability of the catalyst for complex mixtures. The produced water from an oil and gas company has been employed as the real sample whose GC analysis was given in Fig. 9A. The amount of 10 mL real sample undergoes the AOP process using 0.054 g (N, S)-MPC catalyst and 0.27 g sodium peroxydisulfate at 25 °C for 24 h. The GC analysis after the reaction time has been provided in Fig. 9B. As observed in Fig. 9, some peaks of the organic matter have a certain degree of decline, and some peaks even disappear. The total decontamination of 61% was calculated according to the sum of peak integrals in the whole region. We also divided the GC spectrum to three regions containing the retention time of 4–15 min, 15–25 min, and 25–42 min, which the removal was determined to be 96, 56, and 27%, respectively. Interestingly, a high yield of decontamination was obtained for the compounds to pass through the column in the first 15 min, which are probably lower molecular weights. As the retention time increases, the removal efficiency was decreased, which can be related to the larger molecular weight and lower efficiency of the catalyst for these compounds.

**Figure 10:**
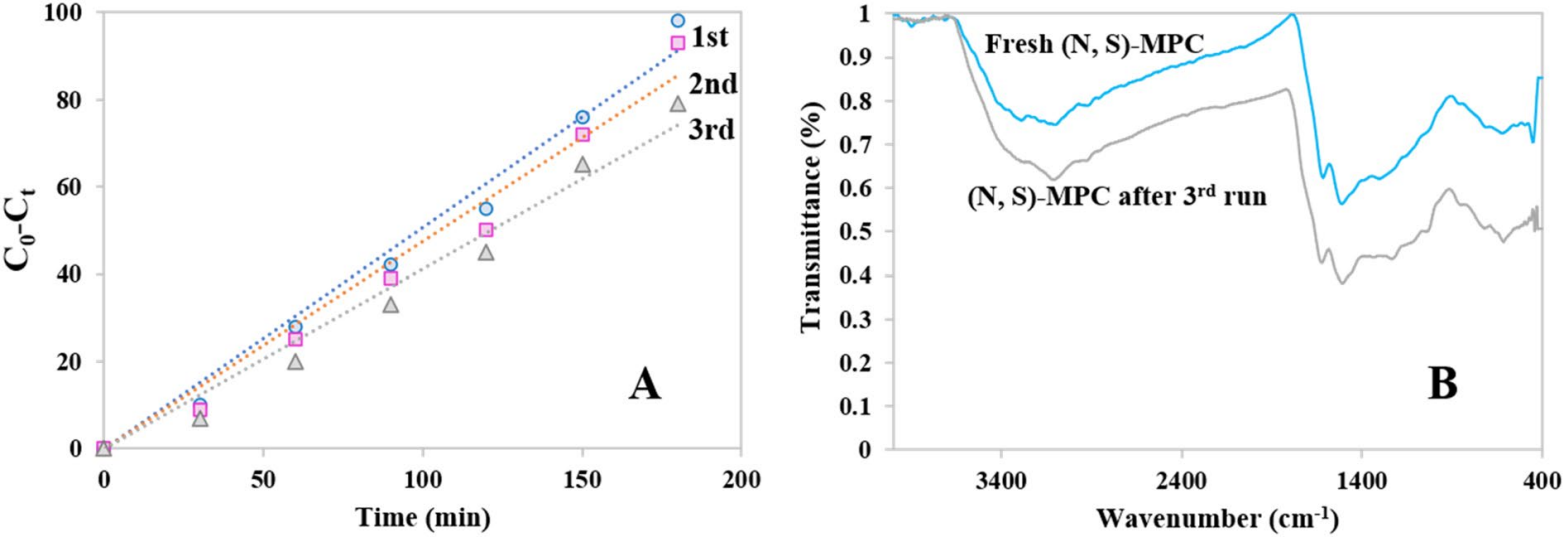
Recyclability tests of (N, S)-MPC for three runs (**A**), FT-IR spectra of fresh (N, S)-MPC, and (N, S)-MPC after three runs (**B**).

Recyclability is one of the advantages and features of heterogeneous catalysts. To evaluate the recyclability of (N, S)-MPC, the experiment was performed for three runs. After each run, the catalyst was removed, washed with DI water, and dried to use for the subsequent run. The recyclability tests of (N, S)-MPC were exhibited in Fig. 10A. The as-prepared (N, S)-MPC showed 98% removal after 180 min. In the 2nd and 3rd run, (N, S)-MPC provided 93% and 79% removal, respectively.

FT-IR analysis was performed for (N, S)-MPC after three runs to study the possible influence of the oxidation reactions on the functionality of as-prepared (N, S)-MPC (Fig. 10B). As observed, the bands remained unchanged which indicated the stability of the catalyst after three runs and its heterogeneity nature. For the next runs, the catalytic efficiency dramatically decreased probably because of the coverage of adsorbed intermediates and the strong oxidation process which have also been observed in previously reported works on the metal-free catalysts for AOP reaction^14,46^. The reactivation process, including thermal treatment and ultrasonication, can be done to remove the adsorbed intermediates and retain the catalytic activity.

## Conclusion

Poor stability and metal leaching are the main drawbacks of metal-based catalytic reactions. In this work, we introduced nitrogen and sulfur co-doped mesoporous carbon material as a highly efficient metal-free catalyst for persulfate-based AOP. The degradation of 4-NP was accomplished as a model reaction to explore the catalytic activity, efficiency, and recyclability. The COD removal was calculated to be 76% using (N, S)-MPC catalyst. Interestingly, the degradations kinetics of 4-NP followed the zero-order kinetics with the rate constant of 0.505 min^-1^. The radical quenching experiment was accomplished to investigate the activation pathway of degradation. Various factors might be responsible for the high activity of (N, S)-MPC, including the porous carbon structure indicating the carbon bridge and electron transfer as well as interaction with the benzene ring, the presence of heteroatoms enhancing the electron transfer, and structural defect. Among the metal-free carbon-based materials for persulfate activation, the straightforward and low-cost synthetic procedure, and low catalyst amount make (N, S)-MPC an excellent choice for practical applications. In addition, the presented catalyst opened a promising research field for biomedical applications in which the absence of metals is highly demanded due to the lack of toxicity and higher biocompatibility including theragnostic nanomedicine.

## Data Availability

All data generated or analyzed during this study are included in this article**.**
